# The Isolation of Culturable Bacteria in *Ixodes ricinus* Ticks of a Belgian Peri-Urban Forest Uncovers Opportunistic Bacteria Potentially Important for Public Health

**DOI:** 10.3390/ijerph182212134

**Published:** 2021-11-19

**Authors:** Raphaël Rousseau, Sophie O. Vanwambeke, Cécile Boland, Marcella Mori

**Affiliations:** 1 Georges Lemaître Centre for Earth and Climate Research, Earth and Life Institute, Université Catholique de Louvain (UCLouvain), Place Pasteur, B-1348 Louvain-la-Neuve, Belgium; sophie.vanwambeke@uclouvain.be; 2 Sciensano, Veterinary Bacteriology, Groeselenberg 99, B-1180 Ukkel, Belgium; cecile.boland@sciensano.be

**Keywords:** *Ixodes ricinus*, microbiota, bacterial flora, *Cedecea davisae*, species diversity

## Abstract

Most bacteria found in ticks are not pathogenic to humans but coexist as endosymbionts and may have effects on tick fitness and pathogen transmission. In this study, we cultured and isolated 78 bacteria from 954 *Ixodes ricinus* ticks collected in 7 sites of a Belgian peri-urban forest. Most isolated species were non-pathogenic environmental microorganisms, and were from the Firmicutes (69.23%), Actinobacteria (17.95%) and Proteobacteria (3.84%) phyla. One bacterium isolate was particularly noteworthy, *Cedecea davisae*, a rare opportunistic bacterium, naturally resistant to various antibiotics. It has never been isolated from ticks before and this isolated strain was resistant to ampicillin, cefoxitin and colistin. Although cultivable bacteria do not represent the complete tick microbiota, the sites presented variable bacterial compositions and diversities. This study is a first attempt to describe the culturable microbiota of ticks collected in Belgium. Further collections and analyses of ticks of different species, from various areas and using other bacterial identification methods would strengthen these results. However, they highlight the importance of ticks as potential sentinel for opportunistic bacteria of public health importance.

## 1. Introduction

Ticks are important vectors of pathogens affecting humans and animals worldwide [1,2,3]⁠. These pathogens attract great public heath interest, and many studies tried to estimate the influence of human, environmental, and climatic factors on tick abundance and pathogen prevalence [4,5,6]⁠. However, tick bacterial composition is not restricted to pathogenic agents. A bigger and richer community of symbiotic, commensal, and parasitic microorganisms coexists in *Ixodes* ticks, forming a complex microbiota. Pollet et al. defined tick microbiota as “the assemblage of all microorganisms present in and on ticks” [4]⁠ (p. 3). Tick microbiota often consists of endosymbionts, engaged in relation with their tick hosts, influencing their fitness, vector capacity and co-infections with pathogenic agents [4,7]⁠. In Wisconsin (United States), male *Ixodes scapularis* ticks had lower rates of *Borrelia burgdorferi* infection when they were infected by rickettsial endosymbionts [8]⁠. Considering the potential importance of microbiota in *Ixodes* ticks and tick-borne pathogens (TBP), as well as the development of new metagenomic approaches, interest has steadily grown in recent years.

*Ixodes ricinus* is the most widespread tick species in Western Europe and is mainly found in forests, parks, and semi-natural habitats [5]⁠. Its presence and abundance are affected by broad-scale characteristics, e.g., temperature, vegetation type or elevation, and fine-scale characteristics, e.g., undergrowth or the presence of specific hosts [9]⁠. Its microbiota is also highly variable between micro-climates, regions, and habitats [7,9,10,11,12,13]⁠. The relative importance of these factors and their interactions on *Ixodes* microbiota is not well understood but is currently attracting a growing interest [10]⁠.

An increasing number of studies on tick microbiota have been published over the past years [14]⁠. Yet, many microorganisms and interactions in tick microbiota are unknown or unidentified [7,15]⁠. Bacteria were classically identified with individual strains cultivated in the laboratory. However, they may be unculturable or difficult to culture. Recently, new molecular metagenomic approaches, such as Next Generation Sequencing (NGS), have been developed. This method has been explored for *I. ricinus* by Carpi et al. [11]⁠ and has expanded since (e.g., [13,16,17]⁠). The very large number of microorganisms identified by NGS suggests that some have an environmental origin and may be unable to survive and develop in ticks [14]⁠. The composition of tick microbiota consists of microbes vertically transmitted or acquired from local environments [18]⁠.

Tick microbiota remains largely unknown but the presence of bacteria of environmental origin may suggest that the local environment influences the presence, abundance, and diversity of bacterial communities in *I. ricinus*. In this study, *Ixodes* ticks were collected by dragging in seven sites in a Belgian peri-urban forest throughout the season of tick activity. These sites represent several aspects of the forests in terms of tree covers and undergrowth. We developed a method to isolate and identify culturable bacteria from ticks, and we analyzed their diversity between different sites and different local environments.

## 2. Materials and Methods

### 2.1. Study Area

**Figure 1:**
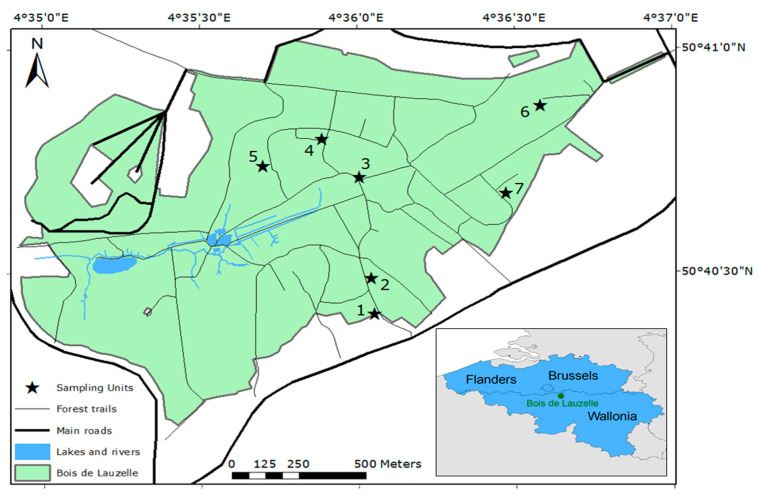
Location of the seven sampling sites in the Bois de Lauzelle.

Ticks were collected at seven locations in the Bois de Lauzelle, a periurban forest of 200 hectares located in the municipalities of Wavre and Ottignies-Louvain-la-Neuve in the Walloon Brabant province of Belgium (Figure 1). The forest is delimited by another forest and a golf facility in the north, and by high-speed roads in the east, south, and west. This private forest is the property and under the management of the neighboring university (UCLouvain) since 1968. The forest has loamy and sandy-loamy soils and is also a Natura 2000 site since 2002 (code BE31006). It has a mean altitude of 103 m (range: 45–153 m) and its hilly topography is influenced by the Blanc-Ry River.

Seven sites were selected for sampling (Figure 1). In each site, we sampled 1 transect of 10 m with a 1 m × 1 m white flannel. The mean distance between two sites is 632 m (range: 146–1080 m). These sites presented diverse characteristics of the forest (Table 1). The western part of the forest was not accessible. Site 1 was at an entrance of the forest, close to a cub scout’s meeting place. The transect was located on a loamy soil covered by grass and dead leaves. Site 2 was deeper in the forest, away from the trails on dense undergrowth of brambles and ferns, and dead leaves. Site 3 was at the junction of two trails, close to a bench and an information panel. The underground vegetation was not high or abundant. Site 4 was at the edge of a small clearing, composed by middle-height grass. Site 5 was in an area where trees had recently been cut. The underground vegetation was poor, mainly composed by a layer of moss. Site 6 was close to a barbecue facility, but away from the trails, with a dense undergrowth composed of brambles and ferns, and dead leaves. Site 7 was at the edge between coniferous and deciduous tree stands, away from the trail and with dense undergrowth.

### 2.2. Data Collection and Preparation

Ticks were sampled in each site every 2 weeks between March and November 2016, by dragging a 1 m^2^-white flannel 10 times over an area of 10 m^2^. Ticks were removed and collected after each drag and stored at −80 °C until processed for DNA extraction. Larvae were not systematically sampled.

Tick life stage and species were identified using a Leica EZ4 binocular (X35), based on two morphological identification keys [19,20]⁠. After identification, ticks were washed in 3 successive baths for two min each, the first one with 70 °C-alcohol to kill bacteria on the surface and the last two with sterilized water. The water was screened to control sterilization. We made culture from the washing water after the ethanol bath resulting in an absence of bacterial isolation. Then, they were individually smashed in Eppendorfs containing 200 μL of Dulbecco’s modified Eagle’s medium (DMEM) cell culture medium. Vortex mixed homogenate of ticks were grouped in pools of four (50 µL/each, for a total volume of 200 µL) based on the site and season of collection. Larvae were pooled by four, separately from nymphs and adults. A total of 100 μL of each pool were placed in culture. Moreover, 50 μL in a selective medium based on Agar bacteriological Amersco and antibiotic (Vancomycin, Polymyxin, Trimethoprim and Cephalothin-with the scope of isolation of *Francisella tularensis*), and 50 μL in a non-selective medium. The remaining 100 µL were used for DNA extraction.

### 2.3. DNA Extraction and High-Resolution Melting Analysis for Ixodes spp. Confirmation

Initially, 5 µL of lysozyme (10 mg/mL) were added to the 100 µL pool medium for DNA extraction. After incubation at 37 °C for 30 min, 200 µL of lysis buffer from the MagMax™ Isolation Kit and 25 µL of proteinase K were included to continue with an incubation at 56 °C for 1 h. The homogenate product was then centrifuged at 11,000 rpm for 3 min and the supernatant processed with the MagMax™ Isolation Kit (Applied Biosystems; Thermo Fisher Scientific, Inc., Waltham, MA, USA) according to the manufacturer’s instructions. Next, 1/10th of the eluted DNA was used to confirm the tick genus by analysis of polymorphisms in 5S and ITS2 genes. This assessment was achieved with the high-resolution melting analysis (HRMA) using a SYBR green based real-time PCR run on a Light cycler^®^ 480 Instrument II (Roche Molecular Systems, Inc., Pleasanton, CA, USA). The cycle run consisted of 1x cycle of 10 min at 95 °C followed by 50 cycles of 8 s at 95 °C, 5 s at 62 °C and 5 s at 72 °C. The last step consisted of a melting curve assessment. The results were expressed as melting temperature of the corresponding amplicon. The DNA extracted from the ticks was available for molecular detection of pathogens and will be the subject of further studies. The DNA of isolated bacterial colonies used for whole genome sequencing (WGS) was obtained with the silica-based column method of the DNeasy Blood & Tissue Kit (Qiagen^®^, Hilden, Germany) following the manufacturer’s instructions.

### 2.4. Bacterial Identification

Once the colony was isolated in hard medium, the bacterium was identified by the Bruker MALDI Biotyper IVD MSP Identification Standard Method 1.1. All colonies present on the plate were collected for identification. Only scores above 2 were considered. Alternatively, ribosomal 16S DNA was amplified by standard PCR and sequenced using universal primers 27F and 1492R [21]⁠ (list of primes in Supplementary Figure S1). One strain was analyzed by WGS and identified with Kraken (Galaxy Version 2.1.1). A representative diagram of the entire workflow is provided in the Figure S1.

### 2.5. Antimicrobial Susceptibility Testing

The minimum inhibitory concentration (MIC) was determined for 20 antimicrobials with the broth microdilution method using EUVSEC and EUST plates (SensititreTM, Thermo Fisher Scientific, Waltham, MA, USA). Following an 18–24 h incubation period, plates were read with a SensititreTM VizionTM instrument (Thermo Fisher Scientific, Waltham, MA, USA) using Sensivision software (MCS Diagnostics BV, Swalmen, The Netherlands). MICs were interpreted according to EUCAST breakpoints defined for Enterobacterales (http://www.eucast.org/clinical_breakpoints, accessed on 1 January 2021) or epidemiological cut-off values (ECOFF).

### 2.6. WGS and In Silico Analysis of Resistance Genes

WGS was performed on MiSeq platform (Illumina) using protocols defined elsewhere [22]⁠. Briefly, short read sequencing libraries were prepared using a Nextera XT kit (Illumina) and sequenced with a 250-bp paired-end protocol (MiSeq v3 chemistry) according to the manufacturer’s instructions. Raw sequencing data of *C. davisae* isolated in this study were submitted to NCBI (https://www.ncbi.nlm.nih.gov/ accessed on 16 September 2021) and are available under the accession number SAMN22562744. For the analysis of genome data, read quality control and trimming were performed with FastQC (Galaxy Version 0.72) and Trimmomatic (Galaxy Version 0.38.0). Genome reference-based assembly was achieved with Bowtie 2 (default settings in Galaxy Version 3.12.0). using the genome of *C. davisae* DSM 4568 (PRJNA30753) as reference. Derived contigs were analyzed in KEGG Automatic Annotation Server (KAAS, https://www.genome.jp/kegg/kaas/ accessed on 16 September 2021) Ver. 2.1, which provided indication on the antimicrobial resistance determinants. De novo assembly coupled to *Resfinder* (Galaxy Version 0.2) and KAAS analyses did not retrieve results on resistance determinants, and it was not further used.

### 2.7. Bacterial Diversity

Bacterial diversity between the sites was estimated based on alpha and beta diversity indexes [23,24]⁠. All of the analyses were performed with the vegan package [25]⁠ in R 3.6.3 [26]⁠. Only pools of nymphs and adults were considered for diversity analyses, as larvae were not systematically sampled. Alpha diversity describes the diversity in each site separately and was measured by three indexes. The first index is the abundance, which is the total number of bacteria isolated in ticks by site, regardless of their species. The second, species richness, is the number of different species of bacteria by site, regardless their abundance. The last, species diversity, incorporates both the number of species and their abundance. It was measured here with the Shannon Diversity Index (H) [27]⁠. H is one of the most commonly used diversity indexes in ecology [28]⁠. It characterizes diversity based on the number of species present and the number of organisms per species. The values vary generally between 1 and 4, with low values reflecting low diversity. The number of ticks by site and alpha diversity indexes were tested for spatial autocorrelation using Moran’s I.

Beta diversity considers differences in diversity between two sites and is represented by the presence or absence of different species between two or more sites [29]⁠. Beta diversity indices were measured here as $\beta_{w}$ from [24]⁠. Since this index may simply increase from an increase in the number of sites sampled, *$\beta_{w}$* was calculated from pairwise comparison of sites (Equation (1)).

(1) $$\beta_{w}=\frac{\frac{\left(a+b+c\right)}{\left(2a+b+c\right)}}{2}-1$$

where *a* is the number of species shared in the two sites, *b*, the number of unique species in the first site, and *c*, the number of species in the second site. Based on this index, we calculated the Sorensen index of dissimilarity, which varies from zero, indicating that the two sites share all their species, to one when the bacterial communities are totally different.

## 3. Results

### 3.1. Description of the Tick Culturable Bacteria

A total of 954 *Ixodes* ticks were collected, 86 larvae, 787 nymphs, 37 males and 44 females (Table 2). All of the ticks captured were Ixodidae. One tick was identified as *Ixodes ventalloi*, 13 as *Ixodes frontalis*, and the remaining as *Ixodes ricinus*. A percentage 8.5% of the ticks were adults. It was relatively constant between the sites, except site 1 with a high percentage of adults (37.9%). Tick abundance was heterogeneous, ranging from sites with relatively low (sites 1, 3, 5 and 6) to high abundances (sites 2, 4 and 7).

**Figure 2:**
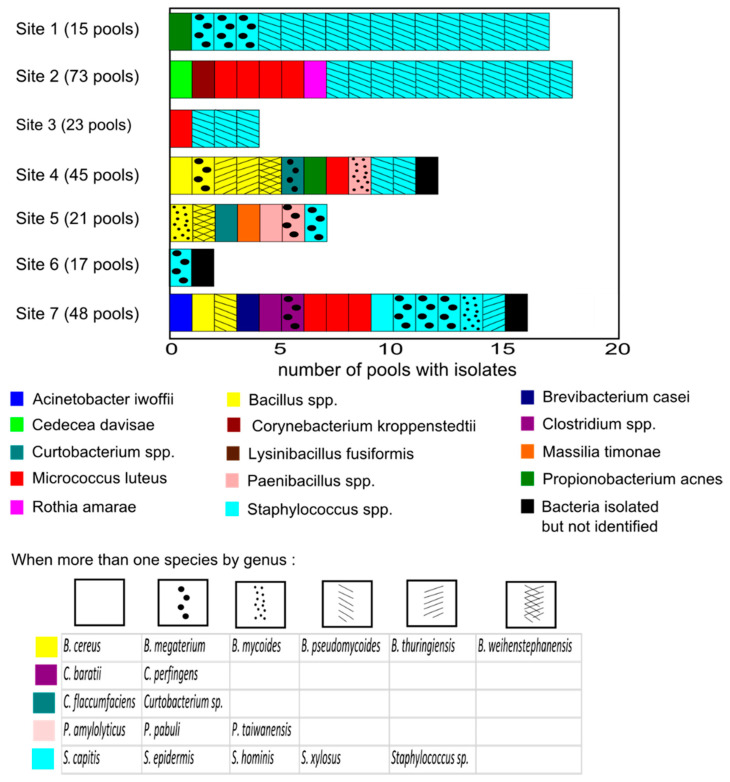
Number of pools with culturable bacterial isolates by site and species. Sometimes more than one species was isolated from the same pool.

Ticks were grouped in 242 pools. A total of 21 pools were made up exclusively of larvae. After culture, PCR amplification and sequencing, 76 strains were isolated from 63 pools. The list of bacteria isolated from the 242 pools is provided in the Supplementary Materials, (Table S1). Moreover, 26 species were isolated (Figure 2). Three bacteria were not identified neither by the MALDI-TOF MS method nor the ribosomal 16S DNA sequencing. Most strains were environmental bacteria, especially with soil or skin-mucosal origin. Except for the three *Proteobacteria*, all identified strains were Gram-positive (68/71). Three culturable bacteria were isolated from pools of larvae: *Bacillus megaterium*, *Micrococcus luteus* and *Staphylococcus hominis.*

**Figure 3:**
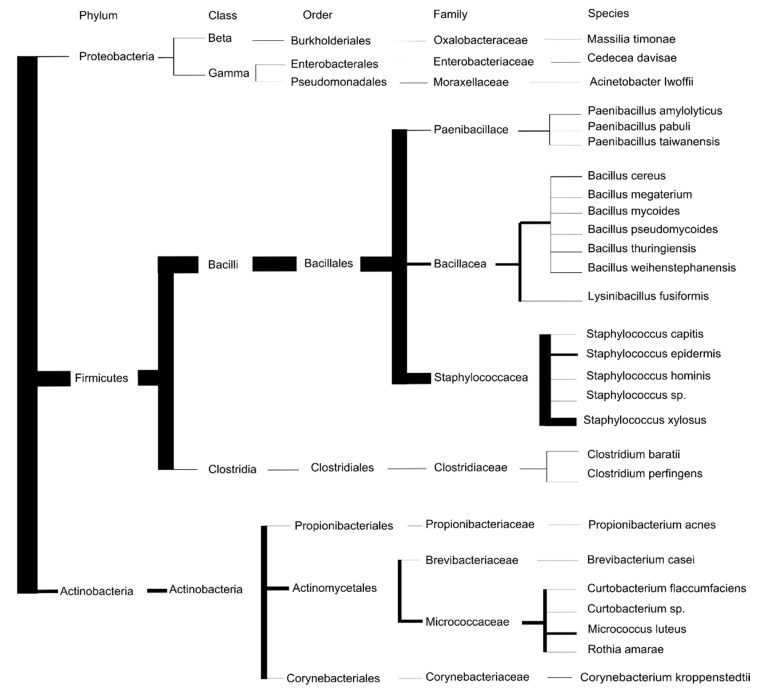
Dendrogram of bacterial species adapted from Bryksin and Matsumura [31]⁠ with permission. Branch lengths do not represent evolutionary distance. Branch widths represent the number of bacteria isolated. Only species related to this study are shown.

Most isolated culturable bacteria were from the Firmicutes phylum (55 isolates from 16 species), mainly from the *Staphylococcus* (40 isolates) and *Bacillus* (9 isolates) genera. *Staphylococcus xylosus* and *Staphylococcus epidermidis* were isolated 29 and 8 times, respectively (Figure 3). *Staphylococcus xylosus* was the dominant strain isolated in the most humid environments (sites 1–3). Other *Staphylococcus* species were *S. capitis* and *S. hominis*. One *Staphylococcus* was not identified. These bacteria are common bacteria of the skin and mucous membranes of humans and animals [30]⁠. Six species of the genus *Bacillus* were isolated: *B. cereus, B. megaterium, B. mycoides, B. pseudomycoides, B. thuringiensis*, and *B. weihenstephanensis*. The last Firmicutes species isolated were *Clostridium baratii*, *Clostridium perfingens*, *Lysinibacillus fusiformis, Paenibacillus amylolyticus, Paenibacillus pabuli* and *Paenibacillus taiwanensis*.

One-fifth of the culturable bacteria were from Actinobacteria phylum (fifteen isolates from six species). Nine *Micrococcus luteus* were isolated, from pools from five sites, making it the second most abundant species. Other Actinobacteria were *Curtobacterium flaccumfaciens, Rothia amarae, Brevibacterium casei, Corynebacterium kroppenstedtii* and *Propionibacterium acnes.* One *Curtobacterium* was isolated but not identified.

Three culturable bacteria species were from the Proteobacteria phylum: *Acinetobacter lwoffii, Massilia timonae*, and *Cedecea davisae.*

### 3.2. Diversity

We did not include the three unidentified bacteria, *Curtobacterium* sp., *Staphylococcus* sp., and the three bacteria isolated from pools of larvae for the diversity analyses (Table 3). A total of 68 bacteria were considered. Sites 1, 2 and 7 presented the highest abundance of bacteria, with, respectively, 18, 17 and 13 isolates. Sites 3 and 6 had the lowest, with 4 and 1 strains detected. The abundance of bacterial isolates by site was not correlated with the number of nymphs and adult ticks (Spearman’s rho = 0.21, *p*-value = 0.66).

The richest sites in bacteria species were sites 7, 5 and 4, with, respectively, 9, 7 and 6 different species isolated, compared to the less rich sites (6, 2, 3 and 1). Sites 7, 5 and 4 were the most diverse (H = 2.06, 1.95 and 1.73, respectively), and sites 3, 1 and 2, the least (H = 0.56, 0.68 and 1.11, respectively). We could not a compute diversity index for site 6, as only 1 strain was isolated. *Staphylococcus xylosus* was abundant in sites with low bacterial diversity (sites 1–3), where they constitute 60–76% of the strains isolated. In site 1, *S. xylosus* was isolated in 86.67% of the pools (13/15). Except in site 4, they were absent from sites with high bacterial biodiversity (sites 5 and 7).

There was no spatial autocorrelation between sites for the number of adult and nymphal ticks sampled (Moran’s I *p*-value = 0.11), percentage of adults (Moran’s I *p*-value = 0.85), abundance (Moran’s I *p*-value = 0.23), species richness (Moran’s I *p*-value = 0.46), and Shannon diversity index (Moran’s I *p*-value = 0.64).

Beta diversity indices from the Sorensen matrix of dissimilarity between the seven sites were high (mean = 0.81, standard deviation = 0.16) indicating that the sites had relatively different communities (Table 4). Sites 2 and 3 were the most similar (index of dissimilarity = 0.43), sharing the 2 most abundant species identified, *S. xylosus* and *M. micrococcus*. Site 6 was the less similar site, especially due to the presence of a single identified isolate: *S. epidermidis*.

### 3.3. Antibiotic Resistance Pattern and Genomic Characteristics of C. davisae

One bacterium was isolated both in selective and non-selective media, evidencing an intrinsic resistance to various antibiotics, *C. davisae*. Its bacterial identification was confirmed by MALDI-TOF MS, 16S rRNA sequencing and WGS. The phenotypic resistance profile of this *C. davisae* strain was characterized through MIC determination for 20 antimicrobials (Table 5). Phenotypic resistance was observed with cefoxitin (MIC of >16 μg/mL), ampicillin (MIC of >64 μg/mL) and colistin (MIC of >16 μg/mL). To gain insight into the molecular features underlying the antimicrobial resistance pattern, WGS data were used to identify orthologs of resistance pathways in KAAS. Within the antimicrobial resistance genes categories, four gene sets were identified: (i) β-Lactam resistance, (ii) vancomycin resistance, (iii) cationic antimicrobial peptide (CAMP) resistance, including the LPS modification system associated with colistin resistance, and (iv) a miscellanea of genes implicated in multidrug resistance phenotype (complete list given in Supplementary Table S2). In this strain, ampicillin resistance is mediated by genes of the mec family, the bla system and the ParR/ParS, CusR/CusS two-component systems. Colistin resistance is associated with lipopolysaccharide (LPS) modification via cationic substitution as the PhoQ/PhoP two-component system is involved. No mcr genes (1 to 10) were found excluding the possibility of acquisition of colistin resistance through horizontal gene transfer.

## 4. Discussion

Studying elements of tick microbiota is important because the bacterial flora may specifically influence tick fitness, reproduction, and competence as vectors. These bacteria may also facilitate or compete with tick-borne pathogens [18]⁠. Bacterial phyla distribution and relative abundances found in this study were consistent with previous publications on culturable bacteria. We found the same proportions of the 3 phyla, Firmicutes, Actinobacteria, and Proteobacteria, in the 113 bacteria isolated from the gut of the haematophagous *Glossina pallidipes* [32]⁠. Six studies analyzed culturable bacteria in Ixodidae ticks, one in *Ixodes scapularis* in the Unites States [33]⁠, one in *Ixodes holocyclus* in Australia [3]⁠, and four in *Ixodes ricinus*, mostly in central Europe [15,34,35,36]⁠. (Supplementary Table S3). Most bacterial genera isolated here had already been identified in ticks elsewhere, such as *Staphylococcus*, *Micrococcus*, *Bacillus*, *Paenibacillus*, *Acinetobacter*, *Propionibacterium*. A deeper comparison at the species level was not possible because of the absence of standard method to analyze culturable bacteria, as different culture media target different bacterial communities.

There is, to our knowledge, no scientific information about the composition of Ixodidae microbiota in Belgium, where ticks were mostly screened for known pathogenic agents (e.g., [37]⁠). This study is a first attempt to describe culturable bacterial composition in ticks from Belgium. We discovered a variable diversity of cultivable bacteria genus from ticks collected from the same forest stand. No bacteria species were found at all sites, and the most abundant, *S. xylosus*, was found in four sites (sites 1–3 and 7). *Staphylococcus xylosus* is a gram-positive bacterium that is generally not pathogenic, although a few strains are related to human infections [38,39]⁠. Another member of the *Staphylococcus* genus found in this study, *S. epidermidis* is an opportunistic pathogen widely spread in the environment and in human skins and mucosal surfaces where it can cause nosocomial infections [30,40].

Forest floor structure is important for tick survival and may affect tick microbial composition [9]⁠. For example, soil bacterial communities are very diverse, and even if carbon mineralization rate can explain abundances of specific phyla, the ecological mechanisms explaining this diversity are not fully understood yet [41]⁠ The seven sites presented contrasted bacterial diversities in ticks, as indicated by the Sorensen matrix of dissimilarity (Table 5). The more similar sites with the more similar bacterial communities (Site 1 versus Site 3, Site 2 versus Site 3, and Site 1 versus Site 6) were not neighbors. No spatial autocorrelation was found for the microbial diversity. However, the presence of many isolates of *S. xylosus* in sites 1,2 and 3, relatively close to each other, potentially suggest a localized environmental source. Sites 4, 5 and 7 had more abundant and diverse bacterial communities, but no relation with vegetation cover or the type of soils was highlighted, probably due to the small number of sites sampled. Although the number of bacterial strains isolated by site were not correlated with the number of ticks sampled, our indices of biodiversity must be moderated by the number of pools of ticks screened by site.

One of the Proteobacteria isolated from site 2*, Cedecea davisae* was particularly noteworthy because a common clinical presentation of infection is bacteremia and it is inherently resistant to antibiotics [42,43]⁠. It is a rare opportunistic bacterium, one of the five species in the genus *Cedecea*, with *C. neteri* and *C. lapagei* [44]⁠. Its isolation in ticks needs to be further investigated to see if it can be transmitted through tick bite. Recent molecular techniques revealed an unexpected bacterial diversity in ticks and indicated that the majority of these bacteria are difficult to culture, or even unculturable [18,45]⁠. The culturable bacteria found in *Ixodes* ticks were typically from four bacterial phyla: Actinobacteria, Bacteroidetes, Firmicutes, and Proteobacteria [46]⁠. Different techniques applied, such as culturing, cloning or species-specific PCR assays, may reveal different taxonomical groups in ticks [9,16]⁠. For example, using metagenomic approaches, the dominant bacteria phyla were Proteobacteria, Actinobacteria and Firmicutes [11]⁠. Proteobacteria was the dominant phyla detected in ticks by sequencing with an Illumina MiSeq machine, with 4 other phyla, Actinobacteria, Bacteroidetes, Firmicutes, and Spirochaetes represented by less than 5% of the total microbiota [47]⁠. The notion of culturable bacteria is also changing through time, as the culturing techniques evolve [48]⁠. Techniques subject to high-throughput sequencing are useful but may overestimate the number of members of the tick microbiota by including many contaminating DNA from environmental sources [2,9,14]⁠. Cultures are therefore a useful complementary method for illumina techniques to describe the microbiota composition of ticks.

Most studies focus on bacteria detection from entire ticks [4]⁠. However, the main interest is on the microbiota present in the tick gut because it represents the entry point for tick-borne pathogens [7]⁠. Guizzo et al. [41]⁠ found that the microbiota of the tick midgut was less abundant and more diverse than the rich but poorly diverse ovarian microbiota, dominated by *Midichloria* sp. Carpi et al. [11]⁠ also observed variation in the occurrence of bacteria among individual ticks. The present study identified a limited and variable number of bacteria in ticks, that were closely related to those found previously in the tick midgut [41]⁠. Their transmission through tick bites requires further functional studies. Bacteria detected in cultures from ticks may not necessarily be part of the tick microbiota; they might indicate temporal contamination from an environmental source. Some bacteria we identified are also found in plants, soils, or the skin of other animals and could be part of the tick exoskeleton. The detection of culturable bacteria in three larvae pools may reflect contamination. The presence of contamination cannot therefore be excluded but is limited in this study for the following reasons. Tick skin bacteria were probably removed, due to the storage at −80 °C between the samplings and the analyzes. A strict surface washing with ethanol and sterilized water was also performed on each tick before DNA extraction. For further studies, Binetruy et al. [49]⁠ recently indicated that bleach should be used over ethanol for tick washing, as the latter method may impact internal bacterial diversity in metagenomics sequencing-based studies, as DNA can easily stick to tick cuticle. Kmet’ and Čaplová [1]⁠ identified the same species of *Staphylococcus*, *Bacillus*, *Micrococcus* and *Brevibacillus* in ethanol sterilized ticks. The bacteria identified here were with distinct homogeneous prints, which is normally excluded in bacteria isolated from outside the ticks.

The spatial and temporal scales are important in studies of tick microbial communities [4,18]⁠. Interactions between microorganisms and the environment depend on the scale at which we consider these interactions. The density of specific animals, with variable reservoir competences, is also important to understand variations in tick microbiota at the local scale [50]⁠. Differences in microbial communities between large areas, climatically and environmentally varied is not surprising. However, in this study, ticks were sampled from seven sites, distant of 632 m on average, in the same forest stand. Wildlife movements are not limited in this forest, which presents contrasted micro-climates in relation to different flora composition and topography. The sites sampled had contrasted culturable bacterial communities.

## 5. Conclusions

It is now clear that TBP are not the only microorganisms present in *Ixodes* ticks, which harbor larger, and still poorly understood microbiota communities. They are particularly important in the TBP complex system as they may influence tick fitness and behavior and interact with other pathogenic and non-pathogenic agents. Recent identifications of tick microorganisms based on several methods and technologies, such as NGS, identified an increasing number of bacteria. It remains questionable if these bacteria are part of tick microbiota or environmental contaminant, unable to physiologically survive and develop in ticks. Using cultures of ticks from the same forest stand, we found complex and variable bacterial communities.

The culturable bacteria found in this study were variable across the seven sites, but consistent with those found in the literature for *Ixodes* ticks. Bacteria from the Firmicutes phylum were the most abundant, followed by the Actinobacteria and the Proteobacteria phyla. Most strains were only isolated once or two times, except *S. xylosus*, *S. epidermidis* and *M. luteus*. The isolation of a strain of *C. davisae* naturally resistant to antibiotics was surprising as it has never been isolated from ticks before, and the transmission of this bacteria through tick bite needs confirmation from other studies. It may suggest a potential tick-mediated transmission of this opportunistic pathogen. The identified culturable bacteria here did not represent the complete tick microbiota, and some isolates might originate from environmental sources, even if this risk was limited with prior washings. It was the first attempt to describe a part of the tick microbiota in Belgium. The bacteria identified were consistent with the literature, but the results need to be confirmed with ticks from other areas, other species, other cultures, and other bacterial identification methods. This information is crucial to help understanding tick-bacteria interactions and detecting the presence of new bacteria in tick microbiota.

## Figures and Tables

**Table 1:** Characteristics of the seven sites sampled.

Sites	Latitude (Degrees)	Longitude (Degrees)	Forest Type	Soil Vegetation	Soils
Site 1	4.6021	50.6729	Deciduous	Grass	Loamy
Site 2	4.6018	50.6742	Deciduous	Brambles	Loamy-Sand
Site 3	4.6014	50.6781	Deciduous	Grass	Loamy
Site 4	4.5994	50.6794	Coniferous	Grass	Sandy
Site 5	4.5963	50.6784	Deciduous	Moss	Sandy
Site 6	4.6111	50.6807	Deciduous	Brambles	Sandy
Site 7	4.6089	50.6772	Coniferous	Brambles	Loamy-Sand

**Table 2:** Number of ticks collected by life stage, sex, and site.

Site	Larvae	Nymphs	Females	Males	Ticks
Site 1	0	36	19	3	58
Site 2	23	248	10	12	293
Site 3	10	71	4	1	86
Site 4	38	131	3	6	178
Site 5	12	66	2	2	82
Site 6	0	60	1	5	66
Site 7	3	175	5	8	191
Total	86	787	44	37	954

**Table 3:** Species diversity indices of the bacterial communities in ticks by sites. Pools made up of larvae were excluded. H refers to Shannon Diversity Index, and NA to not available.

Sites	Pools Tested	Species Richness	Abundance	H
Site 1	15	3	17	0.68
Site 2	69	5	18	1/11
Site 3	20	2	4	0.56
Site 4	35	6	8	1.73
Site 5	18	7	7	1.95
Site 6	17	1	1	0
Site 7	47	9	13	2.06

**Table 4:** Sorensen matrix of dissimilarity.

	Site 1	Site 2	Site 3	Site 4	Site 5	Site 6	Site 7
Site 1	-						
Site 2	0.75	-					
Site 3	0.60	0.43	-				
Site 4	0.78	0.82	0.75	-			
Site 5	0.80	1	1	0.85	-		
Site 6	0.50	1	1	1	0.75	-	
Site 7	0.83	0.86	0.82	0.87	0.88	0.80	-

**Table 5:** MIC (μg/mL) values for the tick-derived C.davisae isolate as defined with the microdilution method Interpretation is based on clinical breakpoints defined by EUCAST (http://www.eucast.org/clinical_breakpoints accessed on 1 January 2021) or ECOFF (indicated by asterisks). Int. stands for interpretation, R. for resistant and S. for sensitive.

Antibiotic Class	Antibiotic Abbreviation	Antibiotic	*Cedecea davisae* (Tick)		*Escherichia coli* ATCC 25922		*Enterobacterales* (EUCAST Clinical Breakpoints 1 January 2021) and ECOFF (*)	
			MIC (µg/mL)	Int.	MIC (µg/mL)	Int.	S≤	R>
Aminoglycosides	GEN	Gentamicin	≤0.5	S	≤0.5	S	2	2
	STR	Streptomycin	≤4	S *				>16 *
Carbapenem	MERO	Meropenem	0.12	S	≤0.03	S	2	8
Cephalosporins	FOT	Cefotaxime	≤0.25	S	≤0.25	S	1	2
	FOX	Cefoxitin	>16	R *			8 *	8 *
	TAZ	Ceftazidime	≤0.5	S	≤0.5	S	1	4
Diterpenes	TIA	Tiamulin	>4					
Fluoroquinolones	CIP	Ciprofloxacin	≤0.015	S	≤0.015	S	0.25	0.5
	NAL	Nalidixic Acid	≤4	S *	≤4	S *		>8
Macrolides, lincosamides and streptogramins	AZI	Azithromycin	16		4			
Penicillins	AMP	Ampicillin	>64	R	4	S	8	8
Tetracyclines	TET	Tetracycline	≤2	S *	≤2	S *		>8 *
	TGC	Tigecycline	≤0.25	S *	≤0.25	S *	1	>0.5
Miscellaneous agent	CHL	Chloramphenicol	≤8	S	≤8	S	8	8
	COL	Colistin	>16	R	≤1	S	2	2
	KAN	Kanamycin	≤4					
	MUP	Mupirocin	256					
	RIF	Rifampicin	>0.5					
	SMX	Sulfamethoxazole	>1024		64			
	TMP	Trimethoprim	≤0.25	S	0.5	S	4	4

* when no clinical breakpoints available, interpretation was based on epidemiological cutoffs (ECOFF) values.

## Data Availability

The data that support the findings of this study are available on request from the corresponding author.

## Supplementary Materials

The following are available online at https://www.mdpi.com/article/10.3390/ijerph182212134/s1, Figure S1. Melting curves derived from analysis of the 5S and ITS2 sequence polymorphisms in *Ixodes* and *Dermacentor* spp.by HRMA. Derivative fluorescent emission data recorded during the melting step. Two main profiles were observed with different Tms, the *Ixodes* spp. group (dark green and brown lines) and the *Dermacentor* spp. group (green, light blue and fuchsia lines). Primers designed for the analysis: primers on 5S for *Ixodes ricinus*: FW: gtcgtagccttccgtcagtc, RV: acggcattcccctactggat; primers on ITS2 for *Dermacentor* spp: FW: cggacacctgcagggaaag, RV: cttccgactctctcgcaaac. Table S1. Pools tested for the isolation and identification of cultivable bacteria by site, season, and tick development stage. Table S2. List of resistance genes and of their orthologs present in the tick-derived *C. davisae* genome as defined with the KAAS analysis. Table S3. Previous scientific publications describing culturable bacteria in *Ixodes* ticks. T. stands for ticks, L. for larvae, N for nymphs, A for adults, M for males and F for females. NA stands for not available.
