# Supplementary material for: The Isolation of Culturable Bacteria in Ixodes ricinus Ticks of a Belgian Peri-Urban Forest Uncovers Opportunistic Bacteria Potentially Important for Public Health
Source: Int J Environ Res Public Health. 2021 Nov 19;18(22):12134. doi: 10.3390/ijerph182212134 (PMC8625411; doi:10.3390/ijerph182212134)
Supplement: Supplementary file 1 [file ijerph-18-12134-s001.zip › ijerph-1405748-supplementary.pdf]

Article

The isolation of culturable bacteria in *Ixodes ricinus* ticks of a Belgian peri-urban forest uncovers opportunistic bacteria potentially important for public health.

Supplementary Materials:

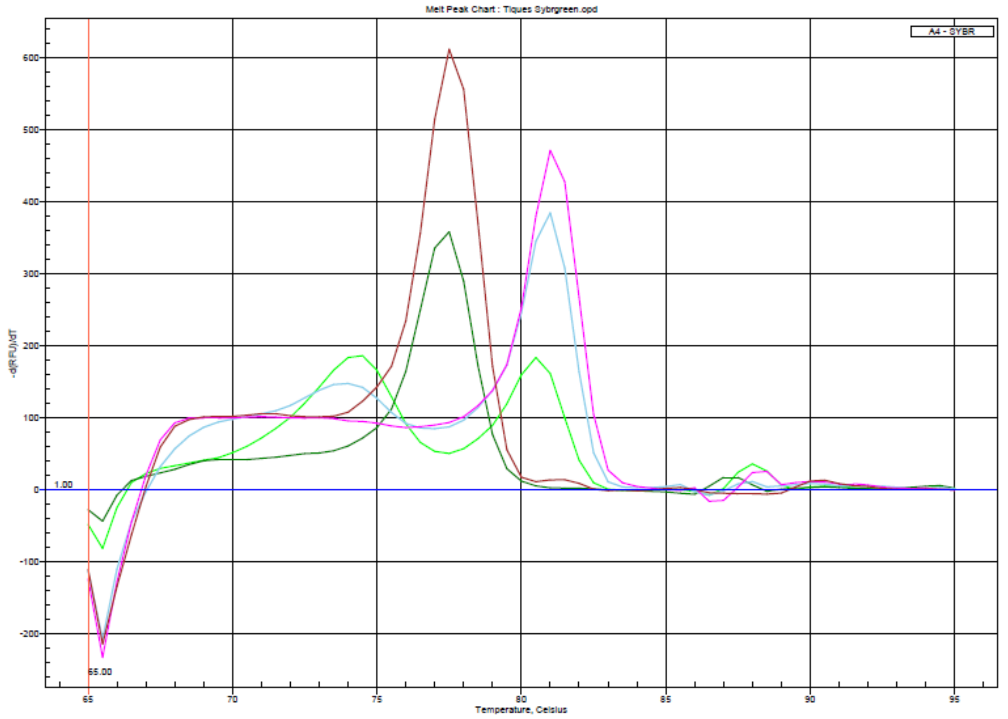

Figure S1. Melting curves derived from analysis of the 5S and ITS2 sequence polymorphisms in *Ixodes* and *Dermacentor* spp.by HRMA. Derivative fluorescent emission data recorded during the melting step. Two main profiles were observed with different Tms, the *Ixodes* spp. group (dark green and brown lines) and the *Dermatocentor* spp. group (green, light blue and fuchsia lines). Primers designed for the analysis: primers on 5S for *Ixodes ricinus:* FW: gtcgtagccttccgtcagtc, RV: acggcattcccctactggat; primers on ITS2 for *Dermacentor* spp: FW: cggacacctgcagggaaag, RV: cttccgactctctcgcaaac

Table S1. Pools tested for the isolation and identification of cultivable bacteria by site, season, and tick development stage. Excel file in the online material.

Table S2. List of resistance genes and of their orthologs present in the tick-derived *C. davisae* genome as defined with the KAAS analysis.

Resistance	Resistance genes encoded in *C. davisae* genome	Orthologs
**β-Lactam**		
Methicillin	mecR1; methicillin resistance protein	K02547
	mecI; methicillin resistance regulatory protein	K02546
	mecA; penicillin-binding protein 2 prime	K02545
Bla system	blaR1; bla regulator protein blaR1	K02172
	blaI; BlaI family transcriptional regulator, penicillinase repressor	K02171
	blaZ; beta-lactamase class A BlaZ	K18766
	penP; beta-lactamase class A	K17836
Imipenem	ParR/ParS two-component system	K18073/ K18072
	CusR/CusS two-component system	K07665/ K07644
	mexT; LysR family transcriptional regulator	K18297
	oprD; imipenem/basic amino acid-specific outer membrane pore	K18093
**Vancomycin**		
D-Ala-D-Ser type	vanRE/vanSE; two-component system, OmpR family	K18349/K18350
	vanT; serine/alanine racemase	K18348
	vanC; D-alanine---D-serine ligase	K18856
	vanXY; D-alanyl-D-alanine dipeptidase/carboxypeptidase	K18866
D-Ala-D-Lac type	vanRB/vanSB; two-component system, OmpR family	K18344/K18345
	vanY; D-alanyl-D-alanine carboxypeptidase	K07260
	vanW; vancomycin resistance protein	K18346
	vanRAc/vanSAc; two-component system, OmpR family	K18352/K18351
	vanK; vancomycin resistance protein VanK	K18354
	vanJ; vancomycin resistance protein VanJ	K18353
	vanH; D-specific alpha-keto acid dehydrogenase	K18347
	vanB; D-alanine---(R)-lactate ligase	K15739
	vanX; D-alanyl-D-alanine dipeptidase	K08641
Other	mgrA; MarR family transcriptional regulator, multiple gene regulator MgrA	K18906
	tetB; MFS transporter, DHA2 family, metal-tetracycline-proton antiporter	K08168
**CAMP**		
dltABCD operon	graR:grasS; two-component system, OmpR family	K19078/K19077
	dltA; D-alanine--poly(phosphoribitol) ligase subunit 1	K03367
	dltB; membrane protein involved in D-alanine export	K03739
	dltC; D-alanine--poly(phosphoribitol) ligase subunit 2	K14188
	dltD; D-alanine transfer protein	K03740
	mprF, fmtC; phosphatidylglycerol lysyltransferase	K14205
VraFG transporter	vraF/vraG; cationic antimicrobial peptide transport system ATP-binding protein	K19079/ K19080
protease PgtE	PhoP/PhoQ two-component system, OmpR family	K07660/K07637
**Miscellanea**		
Efflux pumps	MexAB-OprM, MexCD-OprJ,,MexEF-OprN, MexJK-OprM, MexXY-OprM, MexPQ-OpmE, AdeABC, AcrEF-TolC, MdtEF-TolC, BpeEF-OprC, AbcA, NorB, QacA, MepA, repression of porin OmpF	K18131/ K03585/ K18138/ K18139/K18294/ K18295/ K18296/ K08721/ K18297/ K18298/ K18299/ K18300/ K18301

Table S3. Previous scientific publications describing culturable bacteria in *Ixodes* ticks. T. stands for ticks, L. for larvae, N for nymphs, A for adults, M for males and F for females. NA stands for not available.

**Year(s) (seasons)**	**Sites (country)**	**Bacteria**	**Number of ticks**	**Tick Species**	**Source**
2012 (NA)	Six sites (Hungary)	29 genera, including *Staphylococcus, Micrococcus, Bacillus, Oceanobacillus, Corynebacterium, Propionibacterium, Streptococcus, Streptomyces, Pseudomonas, Sphingomonas*	52 T. (45 L., 19 N., 8 A.)	*I. ricinus*	Egyed and Makrai (2014)
< 1998 (Spring, Summer)	Beltsville, MD (United States)	Seven *Bacillus* species including *B. thuringiensis, B. brevis, and B. sphaericus), Corynebacterium pseudodiptheriticum, Kluyvermonas ascorbata*	43 T. (16 N., 27 A.)	*I. scapularis*	Martin and Schmidtmann (1998)
< 2003 (NA)	NA (Australia)	*Stenotrophomonas, Staphylococcus, Pseudomonas, Acinetobacter, Bacillus.*	21 T. (5 Ixodes)	*I. holocyclus*	Murrell et al. (2003)
< 2012 (Spring, Summer)	Forest region in Silesia (Poland)	*Acinetobacter lwoffi, Pseudomonas ″uorescens, Aeromonas hydrophila, Achromobacter denitrificans, Alcaligenes faecalis, Stenotrophomonas maltophilia, Pseudomonas oryzihabitans, Micrococcus spp., Kocuria varians, Staphylococcus lentus, Kocuria kristinae, Streptococcus pneumoniae, Rhizobium radiobacter, Staphylococcus xylosus.*	36 T. (27 F., 9 M.)	*I. ricinus*	Okła et al. (2012)
2006, 2007 (Spring, Autumn)	Valtice, Obora Soutok and Havraníky (Czech Republic)	- predominantly: *Bacillus* and *Paenibacillus*.<br>- frequent: *Arthrobacter, Corynebacterium, Frigoribacterium, Kocuria, Microbacterium, Micrococcus, Plantibacter, Rhodococcus, Rothia,* and *Staphylococcus*<br>- less frequent: *Advenella, Pseudomonas, Rahnella, Stenotrophomonas, Xanthomonas.*	42 T. (5 L., 10 N., 14 M., 13 F.)	*I. ricinus*	Rudolf et al. (2009)
2003, 2004 (Spring)	Six mixed woodlands (Poland)	*Pasteurella Pneumotropica / haemolytica, Pantoea agglomerans, Serratia marcescens, Serratia plymuthica, Aeromonas hydrophila, Burkholderia cepacia, Chromobacterium violaceum, Pseudomonas aeruginosa, Stenotrophomonas maltophilia*	372 T. (149 N., 101 F., 122 M.)	*I. ricinus*	Stojek and Dutkiewicz (2004)
